# Reliability of a novel point of care device for monitoring diabetic peripheral neuropathy

**DOI:** 10.1038/s41598-023-45841-6

**Published:** 2023-11-03

**Authors:** W. Grabowska, R. King, S. Roll, I. V. Habermann, S. Hörder, K. Hahn, S. N. Willich, S. Schröder, B. Brinkhaus, J. Dietzel

**Affiliations:** 1 Institute of Social Medicine, Epidemiology and Health Economics, Charité -Universitätsmedizin Berlin, Corporate Member of Freie Universität Berlin, Humboldt-Universität zu Berlin, and Berlin Institute of Health, Luisenstr. 57, 10117 Berlin, Germany; 2 Department of Neurology with Experimental Neurology, Charité-Universitätsmedizin Berlin, Corporate Member of Freie Universität Berlin, Humboldt-Universität zu Berlin, and Berlin Institute of Health, Berlin, Germany; 3 Hanse Merkur Center for Traditional Chinese Medicine at the University Medical Center Hamburg-Eppendorf, Martinistrasse 64, 20251 Hamburg, Germany

**Keywords:** Diseases of the nervous system, Chronic pain

## Abstract

We aimed to assess DPNCheck’s reliability for repeated sural nerve conduction (NC) parameters. This post hoc analysis used data from the randomized controlled ACUDPN trial assessing NC of the *N. Suralis* every eight weeks over a 6-month period in 62 patients receiving acupuncture against diabetic peripheral neuropathy (DPN) symptoms. The reliability of DPNCheck for nerve conduction velocity and amplitude was assessed using intraclass correlation coefficients (ICC) and was calculated using data from single time points and repeated measures design. The results of the NC measurements were correlated with the Total Neuropathy Score clinical (TNSc). Overall, for both nerve velocity and amplitude, the reliability at each measurement time point can be described as moderate to good and the reliability using repeated measures design can be described as moderate. Nerve velocity and amplitude showed weak correlation with TNSc. DPNCheck’s reliability results question its suitability for monitoring DPN’s progression. Given the limitation of our analysis, a long-term, pre-specified, fully crossed study should be carried out among patients with DPN to fully determine the suitability of the device for DPN progression monitoring. This was the first analysis assessing the reliability of the DPNCheck for DPN progression monitoring using data from multiple collection time points.

## Introduction

The most common complication of diabetes is peripheral neuropathy (DPN) affecting approximately 50% of diabetic patients^1^. Moreover, diabetes or a diabetogenic metabolic state are the prerequisites for DPN and thus can be present with a long asymptomatic precursor phase prior to disease onset^2^. Apart from a subgroup of diabetic patients presenting small fiber neuropathy that cannot be measured with neurography, DPN´s slowly progressing nature initially affects the long myelinated peripheral nerves, such as *Nervus Suralis*, leading to their demyelinisation^3^. Demyelination of the peripheral nerves can lead to sensory loss and motor impairment, orthopedic problems, ulceration and, in consequence, to recurring infections and amputation. A combination of early detection, intensified glycemic control, and podiatric care are necessary to prevent complications^2^.

The *N. Suralis* is the most frequently examined sensory nerve and its neurography has a high sensitivity (83%) in detecting DPN^4,5^. This examination can be performed with orthodrome or antidrome neurography, as it has been shown that nerves conduct electrical signals in both directions equally^6^. The technique of orthodrome neurography consists of applying the stimulating electrode behind the *Malleolus lateralis* and registering the signal 12-15cm proximally from the stimulation and laterally from the Achilles tendon^7^.

Novel point of care devices (POCD) that can be used by non-specialized staff, are helping to detect the early peripheral manifestations of DPN by measuring the amplitude of the action potential and the conduction velocity of the sural nerve.

**Figure 1:**
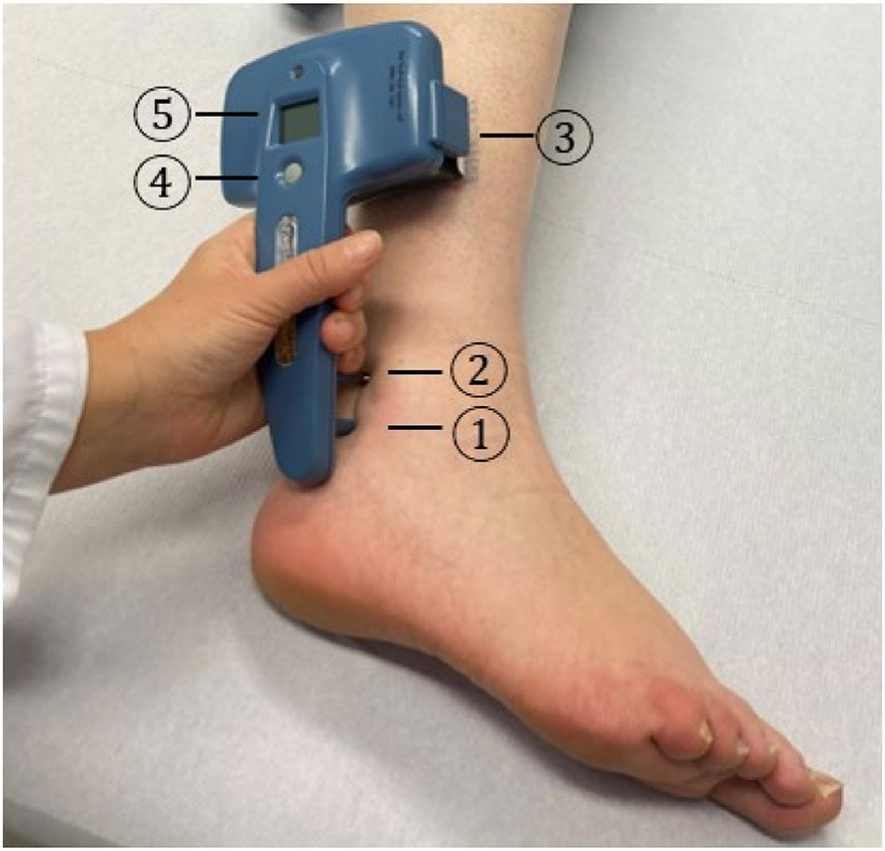
The NC-stat^®^/DPNCheck™ device in situ. 1: infrared thermometer, 2: stimulating probe, 3: biosensor, 4: test button, 5: display screen^8^.

The NC-stat^®^/DPNCheck™ (DPNCheck)^8^ is a POCD designed solely for the automated orthodrome neurography of the sural nerve (Fig. 1). The determination of nerve conduction for both the nerve conduction velocity (NCV) and height of the sensory nerve action potential (SNAP) is fully automatic and does not require any subsequent manual calculations. Results are displayed in m/s and µV for NCV and SNAP, respectively.

Previous studies have shown that the *N. Suralis´* amplitude potential obtained through non-technical staff using the DPNCheck has a high validity when compared to measurements obtained by using the conventional method performed by electromyography technicians^9,10^. The POCD has also been shown to have an excellent inter-rater interclass correlation coefficient (ICC ≥ 0.94) and intra-rater reliability (ICC ≥ 0.79) and high specificity (86.11%) and sensitivity (90.48%) when used to identify DPN^11,12^. Thus far, its reliability has been shown only for supporting the diagnosis of DPN and only one randomized controlled trial (RCT) used the POCD to monitor diabetic neuropathy^13^. Therefore, it remained unclear if its reliability for the purpose of sequential measurements and clinical follow up of DPN progression is adequate.

The objective of this exploratory, post-hoc analysis is to assess the reliability of the DPNCheck device (using repeated measurements of the SNAP and NCV over 16 weeks) and to assess its suitability for clinical follow-up of DPN.

## Methods

### Study design

This post-hoc, exploratory analysis uses data from the clinical RCT “Acupuncture in Diabetic Peripheral Neuropathy—the Randomized, Multicenter ACUDPN Trial,” whose methods have been published elsewhere^14^.

**Figure 2:**
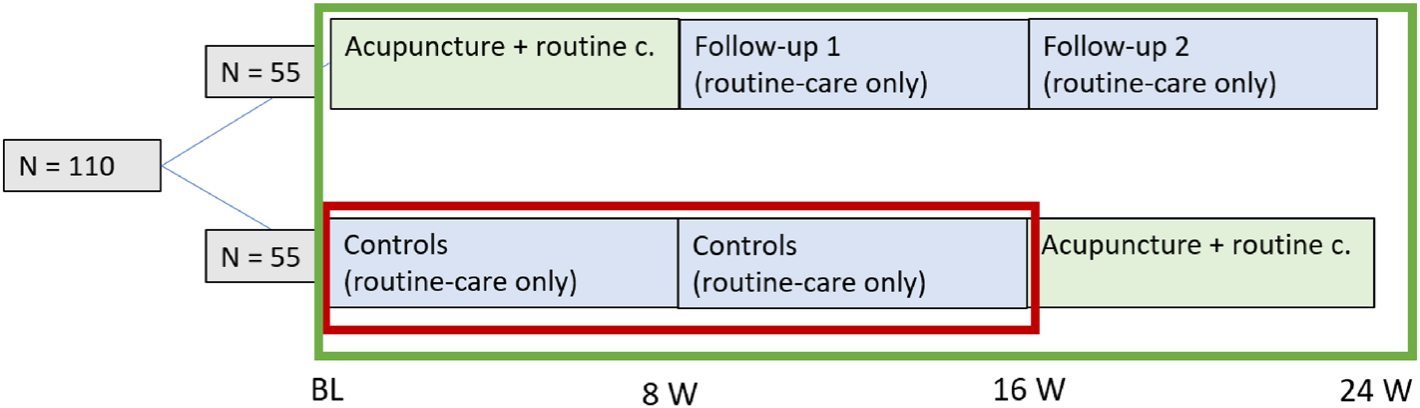
Study Timeline. The within session test/retest reliability assessment will be assessed for each leg at each measurement time point (green rectangle). The subset in red rectangle will be used for the reliability over multiple measurement time points (Baseline BL- 8 weeks -16 weeks).

The ACUDPN study was a two-arm RCT investigating the effect of 12 sessions of acupuncture delivered over 8 weeks in comparison to no treatment in a total of 62 patients aged 18–75 with type II diabetes mellitus and symptomatic DPN in the lower extremities with at least moderate discomfort and pathological baseline values of *N. Suralis* nerve conduction (NC). The control group was a waiting list-group which received acupuncture treatments after the 3rd examination at week 16. Nerve conduction measurements with the POCD of the sural nerve were performed at baseline, and weeks 8, 16, 24 (Fig. 2).

The present analysis addressed the following questions, which were collected together with the planned statistics in a pre-defined analysis plan: What is the test–retest reliability of the POCD within each measurement session obtained for each leg when performed under identical conditions; what is the reliability of the device using the repeated measures and combining data over three sessions—baseline, weeks 8 and 16 for each leg (reliability over time); how often was it necessary to obtain a 3rd measurement during a single session because the POCD returned a technical error (TE) or an amplitude of 0µV and how frequently the first two valid measurements in a given session could be deemed as either a strongly discrepant (Δ > 10m/s; Δ > 2µV) or an implausible result (≥ 60 m/s) regarding the physiological ranges and variability; and how strongly correlated the conduct velocity and amplitude are with the clinical examination results from the Total Neuropathy Score clinical (TNSc).

### Data collection

NC parameters were measured with the handheld POCD DPNCheck^8^, returning NCV (m/s) and the SNAP (µV). Six researchers—four medical doctors and two medical students—from 2 study centers performed the measurements according to their availability in a standardized manner. As a result, JD performed 63% of NC measurements, while the remaining researchers performed between 15 and 3% of the remaining measurements. Within each study session the measurements were performed by one researcher, who followed the instructions for the device handling, especially regarding the exact placement of the POCD, as presented during a supervised training prior to any data collection.

To collect the data patients were placed on their side, with the leg to be examined on top and exposing the calf. A new biosensor mounted on the detecting probe of the POCD was used for every patient. The POCD was then manually programmed to assess the left or right leg of the patient. Electrode gel was applied to the stimulating probe before placing the device on the calf, with the stimulating probe behind the malleolus externus. Having ensured good contact of all the electrodes with the skin, the release button was pushed, and the device would automatically give up to 16 electric discharges of increasing intensity. Values from the nerve conduction were obtained after repeated supramaximal stimulation and noted in the case report file. The procedure was then repeated for the other leg.

The POCD utilizes a linear temperature compensation method for velocity. With the default skin temperature set to 28 °C, the device automatically adjusts the velocity by 1.0 m/s per degree, with a maximum correction of ± 5 m/s. If skin temperature is lower than 23 °C or wrong placement or limb are recognized, the POCD displays a warning. Unlike in the conventional electroneurography (ENG) tools, the height of the participant is not a parameter that can be imputed prior to nerve stimulation, herby it is not accounted for and must be interpreted by the physician.

### Outcomes

The primary outcomes of the ACUDPN study regarding the effects of acupuncture were the DPN related complaints at week 8, measured on a 0–100 mm visual analogue scale, and have been published elsewhere^15^. Gender, age, BMI, and duration of neuropathy were collected at baseline together with other demographical and medical data.

For the outcomes of the present analysis (NC parameters), two valid measurements per patient per leg were performed during each study visit of study participants (detailed procedures described in Sect. “Data collection”). In instances where one of the first two measurements were invalid due to TE or the first two subsequently collected measurements were deemed strongly discrepant (difference > 10m/s or > 2µV) or as implausible (≥ 60 m/s)^7^, a third or fourth measurement per leg was carried out. Additionally, based on the POCD’s manual^8^, a SNAP of 0µV could also be a TE. Thus, such instances were further assessed for feasibility by experts (JD), who determined the validity of SNAP when compared to values of NCV returned by the POCD as stated in the manual^8^.

The TNSc, a composite scale measuring peripheral nerve function and used to assess the results of neurological examination of the lower extremities in a standardized manner. It was evaluated at the same timepoints as the nerve conduction parameters. It has 7 domains, each being scored 0–4 (sensory, pinprick, vibration, motor, autonomic, strength, and deep tendon reflexes). A maximum of 28 points could be achieved, with higher values indicating worse neuropathy. The TNSc was assessed by the participating study neurologists (JD, SS).

### Statistics

Baseline characteristics of study participants relevant for this analysis were analyzed descriptively.

To summarize the collected neurophysiological data, the NC measurements were analyzed descriptively and categorized according to the rules outlined in Sect. “Study design”. Subsequently, the numbers and percentages of measurements deemed as “erroneous,” “implausible,” and “discrepant” were calculated. Additionally, we reported the total number of instances where the collection of a third measurement was required and the number of measurements where SNAP was 0µV and was a plausible result upon experts’ review.

Assuming in the future the POCD would be used by untrained staff following a fixed protocol, and due to uneven progression DPN, the intraclass correlation coefficient (ICCs) were calculated for each leg separately using only the first two measurements per leg, regardless of their ability to capture the nerve conductance signals or categorization, obtained during study sessions.

The test–retest reliability of the POCD at each measurement time point was measured using a one-way random, single measure, absolute agreement ICC.

To calculate the reliability when combining multiple measurement time points (ICC one-way random, single measure, absolute agreement) we utilized the repeated measures design and included only the first three measurement time points (baseline, weeks 8 and 16); since the control group received the intervention between weeks 16 and 24, data obtained at week 24 was excluded from this analysis. To calculate the ICCs, a linear mixed model with fixed and random effects was used^16^. Fixed variables included time, treatment effect, and an intervention interaction on time. Random variables included a (time independent) participant effect, a time session specific participant effect (to account for any possible serial correlation), and a rater effect (to account for rater bias) (see Appendix, Table A1).

Additionally, we conducted a sensitivity analysis to assess the robustness of our findings. Given the limited ability of the DPNCheck device to distinguish between the absence of and a very low nerve amplitude and following the manufacturer’s recommendation to assess for plausibility of any result with an amplitude of 0µV, we conducted a sensitivity analysis, which excluded all the “0” values collected for both the velocity and amplitude of nerve conductance.

The missing data was not replaced. The calculated ICCs were reported with two-sided 95% confidence intervals. ICCs greater than 0.9 were considered to have an excellent reliability^17^.

To assess the relationship between the nerve conductance results with the TNSc, we calculated the correlation of each patient’s session mean velocity and amplitude with TNSc using Pearson correlation.

Confidence intervals and p-values were two-sided. All analyses are considered exploratory (without adjustment for multiple testing).

Analysis was conducted using SAS for Windows, Version 9.4 SAS Institute, Cary, NC, USA) and R, Version 4.1.2 (R Development Core Team). The ICCs were calculated using R and independently confirmed with the ICC9 SAS Macro^18^.

### Ethics approval and consent to participate

The trial was approved by the ethics committee Berlin Germany (Ethikkommission der Charité—Universitätsmedizin Berlin, vote: EA1/183/18) in October 2018. It was performed in compliance with the Declaration of Helsinki and standards of Good Clinical Practice (GCP). Informed written and oral consent was given by all patients prior the beginning of the study. The trial was registered on Clinical-Trials.gov NCT03755960 on the 28/11/2018.

## Results

### Descriptive results

A total of 62 participants (31 per treatment group), were included in the ACUDPN study, with an average age of 68.1 (± 7.43, standard deviation) and 79.0% participants being male (Table 1). The study participants had be diagnosed with diabetes type II on average for 15 years and all but one (98.4%) have been using regular medications to manage their diabetes. The mean velocity (33.1 ± 18.7 m/s) and mean amplitude (3.7 ± 2.1 µV) at baseline were low; an indication of DPN’s presence (Table 1). More information about the study population can be found in the publication reporting the primary results of the study^15^.

**Table 1:** Patients’ baseline characteristics (n = 62).

	*M*ean (± SD) or n (%)
Age	68.09 ± 7.4
Male	49 (79.0%)
Duration of neuropathy	5.3 ± 0.9
BMI > 25	54 (88.5%)
Time since first T2DM diagnosis (years)	15.0 (10.0)
Patients using regularly medications (metformin, insulin) to handle T2DM	61 (98.4%)
Time since T2DM is handled with medication (years)	13.7 (8.8)
HbA1c (mg/dL)	6.5 (0.5)
TNSc	
Left TNSc	10.6 ± 4.1
Right TNSc	10.9 ± 4.0
Conduction velocity (m/s)	33.1 ± 18.7
Right velocity	31.0 ± 19.5
Left velocity	35.4 ± 19.0
Amplitude of potential (µV)	3.7 ± 2.1
Right amplitude	3.9 ± 2.5
Left amplitude	4.0 ± 2.4

*TNSc* total neuropathy score clinical, *T2DMI* Type 2 diabetes mellitus.

In total, 231 patient visits were conducted. The distribution of raters between all study visits was uneven and ranged from 3.0% to 63.0% of conducted study visits.

During the study period, a total of 475 data collection attempts were conducted for both legs together. In 48 (10.1%) of those attempts, the study participant had to be stimulated more than twice to collect the data. Consequently, the data entry from a total of 955 single nerve conduction measurements (single stimulation) were collected (Table 2). Of those single stimulations, 204 (21.4%) were a TE (recorded as “PN” by the POCD). When patients were stimulated for the third time, the POCD returned a valid result only in 3 (6.25%) of the cases.

**Table 2:** Descriptive summary of neurophysiological data (n = 62).

Parameter	N (%)
Measurements included in reliability analysis	
Number of individual measurements used in the reliability analysis:	
Right	398 (49.8%)
Left	401 (50.2%)
Total	799
Total denoted as “implausible” (≥ 60 m/s)	5 (0.62%)
Discrepant measurement between first and second measurement (Δ > 10m/s)	
Right	22 (8.9%)
Left	13 (5.3%)
Total	35 (7.1%)
Discrepant measurement between the first and second measurement (Δ > 2µv)	
Right	16 (6.5%)
Left	12 (4.8%)
Total	28 (5.6%)
Number of measurements where the SNAP was 0µv and was a plausible result	
Right	49 (12.3%)
Left	51 (12.7%)
Total	100 (12.5%)

Following our assumption that the POCD might be used predominantly by untrained staff, only the first two stimulations carried out per each leg per session, regardless of their ability to capture valid data, were included in further descriptive and reliability analysis. Consequently, a total of 799 single data points from both legs were included. Following the definitions of discrepant outcomes, 5 (0.62%) measurements were classified as “implausible” (NCV ≥ 60 m/s) (Table 2). When comparing the results between the two subsequent stimulations obtained during any given session (124 pairs per leg per treatment group), 35 (7.1%) and 28 (5.6%) of the second measurements for NCV and SNAP respectively were classified as “discrepant” (Table 2).

Since the amplitude of 0 µV could be indicative of an erroneous measure, as defined in the manual^8^, such instances were further investigated. Of the 799 single measurements, 100 (12.5%) recorded a value of 0µV (Table 2). In 88 (88.0%) of these the result was consistent with the other measurement obtained during the same session, suggesting that in a patient population with DPN such values are typical; an indication of a pronounced neuropathy, not an error.

### Reliability

Overall, ICCs for the test–retest reliability (within each measurement session) for both nerve velocity and amplitude can be interpreted as moderate to good (ICC range = 0.66–0.91, Table 3)^17^.

**Table 3:** Reliability (ICC) with 95% confidence intervals (CI).

	Test–retest reliability (within session)				Reliability over time
	Week 0	Week 8	Week 16	Week 24	Week 0 to week 16
ICC (95% CI) for velocity					
Right leg	0.89 (0.82, 0.93)	0.66 (0.47, 0.81)	0.79 (0.66, 0.88)	0.81 (0.69, 0.89)	0.50 (0.34, 0.61)
Left leg	0.91 (0.85, 0.94)	0.86 (0.77, 0.92)	0.85 (0.76, 0.92)	0.87 (0.79, 0.93)	0.59 (0.44, 0.69)
ICC (95% CI) for amplitude					
Right leg	0.84 (0.74, 0.90)	0.86 (0.77, 0.92)	0.83 (0.72, 0.90)	0.86 (0.77, 0.92)	0.59 (0.44, 0.70)
Left leg	0.85 (0.76, 0.91)	0.82 (0.71, 0.89)	0.84 (0.73, 0.90)	0.88 (0.80, 0.93)	0.59 (0.45, 0.69)

More specifically, the ICCs for the test–retest reliability of NCV for the left leg were quite high and varied between 0.85 (95% CI 0.76, 0.92) for week 16 and 0.91 (0.85, 0.94) for baseline (Table 3). The ICCs for the test–retest reliability of NCV were slightly lower for the right leg, nevertheless varying between 0.66 (0.47; 0.81) for week 8 and 0.89 (0.82; 0.93) for baseline.

The ICCs for the SNAP showed much less variation for individual legs, as well as overall. The ICCs for SNAP varied between 0.83 (0.72, 0.90) and 0.86 (0.77, 0.92) for right leg and between 0.82 (0.71, 0.89) and 0.88 (0.80, 0.93) for left leg.

The sensitivity analysis excluding the values of “0” for NCV and SNAP yielded, on average, slightly lower ICCs for both NCV and SNAP (Appendix, Table A3). The ICCs obtained in the sensitivity analysis could be still interpreted as providing moderate to good reliability^17^.

For the reliability over time, various models were selected based on AIC minimization (Appendix, Table A2). The session-specific participant effect is quite large, indicating that within each session the values for each participant tend to be highly correlated compared to other sessions, even after taking into account a time-independent participant effect (***α***_***1***_), any possible time trends (***β***_***1***_***, β***_***2***_) and temperature adjustment (for velocity). This could indicate that device placement, or other unknown session specific factors could impede the reliability. The ICCs, ranging from 0.50 (0.34, 0.61) for velocity in the right leg to 0.59 (0.44, 0.70) for amplitude of the left leg (Appendix, Table A2) could be described as moderate^17^.

The sensitivity analysis excluding all ‘’0’’ values yielded quite different results (Appendix, Table A3), suggesting the need to carefully review and interpret such measurements on a case-by-case basis.

### TNSc and measures of nerve conduction

The Pearson correlation shows weak, negative correlations between the nerve conductance parameters and the TNSc values (Table 4). Moderate correlations were also observed amongst the nerve parameters recorded for the same leg. The above results could indicate that the nerve conductance parameters and TNSc capture different/independent hallmarks of DPN disorder.

**Table 4:** Pearson correlations of TNSc and nerve conduction parameters for each leg.

	TNSC RL	TNSC LL	Velocity RL	Velocity LL	Amplitude RL
TNSC RL	–	–	–	–	–
TNSC LL	0.95	–	–	–	–
Velocity RL	− 0.33	− 0.33	–	–	–
Velocity LL	− 0.34	− 0.34	0.54	–	–
Amplitude RL	− 0.33	− 0.32	0.66	0.46	–
Amplitude LL	− 0.24	− 0.27	0.47	0.65	0.54

*RL* right leg, *LL* left leg.

## Discussion

### Summary of results

Although our results support POCD’s good test–retest reliability for NC measurements among patients with moderate to severe DPN due to type II diabetes, with only moderate reliability over time we question the use of the device to monitor the progression of DPN.

More specifically, based on the descriptive analysis, only 0.6% of all measurements were classified as “implausible” (with NCV ≥ 60 m/s) and (7.1%) were classified as strongly discrepant with respect to the preceding measure (Δ > 10 m/s; Δ > 2 µV). Our data showed the device produced a result with a technical error in 20 percent of all the undertaken measurements, indicating that to obtain a meaningful value some study participants had to be electrically stimulated multiple times, which could cause a discomfort or a painful sensation.

Conversely, when a numerical result could be achieved, the POCD was shown to have a moderate to good test–retest reliability for both NCV and SNAP, but only moderate over time reliability. With only 16 weeks between the measurements used in the combined time analysis, the pronounced increase in variability and, thus decrease in reliability, cannot be attributed to the progressive worsening of DPN^19^. Moreover, given the slow nature of Sural nerve’s axonal atrophy and demyelination^19^, a trend indicating a progression of neuropathy over 16 weeks was neither expected nor observed. Rather the sources of the increased variability may stem from the POCD’s usage procedures, such as random changes in the POCD’s placement on the leg between the sessions, or from the device itself.

The sensitivity analysis excluding all null values, yielded consistently lower test–retest reliability ICCs for both NCV and SNAP, but for the reliability over time the results were less consistent.

The ICCs from the sensitivity analysis for reliability over time displayed no trends and remained inconclusive. However, given that in the population of patients with moderate to severe DPN almost 25% of all single measurements displayed a value of zero for either NCV or SNAP, we can infer that (i) in such a sick population these values are not rare and should be expected, and thus (ii) further investigation into the reliability but also validity of the device is needed to determine the value’s plausibility and meaning.

The TNSc results did not correlate strongly with the neurophysiological results, but the two methods do not contain the same amount of objectivity, since the TNSc depends on the patient’s discretion and feedback in most of its elements, and thus it´s precision might be lower.

### Strengths and limitations

DPN is one of the most frequent comorbidities among patients with an impaired glucose tolerance and with diabetes mellites. Thus, a thorough assessment of the reliability of this POCD is of great interest and importance to neurologists, diabetologists, and patients with early stages or with manifested peripheral neuropathy. Our study was the first one conducted in a population of patients with diagnosed DPN and already experiencing mild to severe symptoms related to polyneuropathy, thus the first one investigating the use of this POCD as a DPN’s monitoring device.

Our study was also the first one to evaluate the reliability of sequential measurements collected at 4 different timepoints, over 24 weeks of follow up using this POCD. The analysis included 955 single measurements collected from 62 patients in two different clinical settings by six medical professionals with different levels of experience, capturing the reliability of the device in a setting closely mirroring the medical care provided in the real world. Our results showed good to very good test–retest reliability within each measurement session, but only moderate reliability over multiple time points, suggesting that this POCD might not be suitable for long term monitoring of DPN progression.

Since the primary aim of the ACUDPN trial was not to test POCD’s reliability^14^, the main limitation of this analysis stems from the study design. Our analysis was carried out in a data set with an uneven and not fully crossed distribution of raters per patient. Despite adjusting for raters and treatment effects in the model, our analysis would be more conclusive should we have been able to account for those effects and their potential bias at the level of study design. Further, since we have no comparable gold-standard measurements, we did not measure the POCD’s validity and therefore could not access any positive or negative bias of the collected NC measurements.

Secondly, despite the device being easy to use and requiring minimal training of medical staff prior to its utilization^8,12^, we recorded a rather high number of erroneous measurements (20%). The potential reasons behind them could include an already deteriorated state of the *N.Suralis* due to DPN or the imprecision of the device or of testing procedures. Thus far, no other studies report the rates of erroneous measurements for this POCD, leaving us unable to compare and interpret our rates against other studies or patients’ populations.

Further study limitations stem from the inclusion and exclusion criteria of the ACUDPN trial. Since ours was an exploratory analysis, we are unable to address the reliability of the DPNCheck device in obese subjects (BMI > 35), in children or in patients with diabetes type I.

To address the design limitations, determine the validity and the definite sources of the erroneous measurements, the POCD’s reliability and validity should be tested over time against gold-standard neurography devices, preferably among patients with and without DPN diagnosis and utilizing a fully crossed design (in terms of raters). To determine the impact of training and standard operating procedures on the study results both highly trained and unspecialized medical personnel should collect the data for each patient within each session.

Despite limitations, our research contributes substantially to the body of literature necessary to assess the suitability of the device for DPN diagnosis and monitoring in clinical care.

### Comparison with other studies

A high test–retest reliability of the device among patients with diabetes was first reported by Lee^12^. However, our test–retest reliability, although still indicating a good reliability, are lower than those reported by Lee (ICC of 0.94 for NCV and 0.97 for SNAP)^12^. This difference might be related to our study population, which included subjects with already diagnosed neuropathy and mild to moderate neuropathy symptoms, while only 54% of patients in the study by Lee et al. had polyneuropathy. Our test–retest reliability, however, matched those reported by Scarr et al.^20^, who obtained an ICC of 0.77 and 0.70 for SNAP and NCV respectively in a population of older, type I diabetics, further suggesting that the severity of neuropathy may impact the reliability of the device, a relationship that should be investigated^20^. Of note, there has been no published reliability study for the POCD in the healthy controls, however studies have consistently reported high sensitivity and specificity for the POCD measured among health controls as well as patients with all stages of diabetes and DPN^12,21^

Another reason why our test–retest reliability was lower could stem from the data cleaning processes. Having assumed that in the future the POCD would be used by untrained staff and due to unevenly progressing DPN, the reliability was calculated for each leg separately using only the first two measurements per leg, regardless of their ability to capture the nerve conductance signals. Although likely to increase the external validity of our results, the above assumptions might have led to a decreased reliability observed in the study.

To our knowledge no evaluation of reliability of the sequential measurements over multiple time points with this POCD has been published. However, important aspects should be mentioned here from publications regarding the ICC measurement of *N.Suralis* with non-automated technology that is used in the neurography of all peripheral nerves.

A multicenter trial assessing the reproducibility of NC measurements of various nerves among patients with diabetic polyneuropathy collected two measurements per nerve per time point with a time interval of 1–4 weeks from 172 patients and 132 healthy controls. In the study the SNAP varied most, followed by NCV with an ICC of 0.69 and 0.77 for Sural SNAP and NCV respectively^22^. Both results showing higher reliability over time than our study, yet the authors questioned the use of Sural SNAP for sequential trials^22^.

Another clinical trial on 110 healthy and 46 diabetic patients investigating variability between measurements on 2 consecutive days considered the influence of body height and limb temperature in Sural NCV^23^ [Claus et al.]. The reproducibility analysis showed that Sural NCV and SNAP were significantly influenced by temperature changes and a decrease of 1.6 m/s per 10 cm increase in height was found^23^ [Claus et al.]. Both of those studies point towards the need to develop and standardize the use of more reliable instruments for neurography, which could also account for body temperature and height. The tested POCD does not quite fulfill the above criteria, as it does not account for height and offers only a limited linear temperature adjustment (1.0 m/s per degree, max. correction of ± 5 m/s).

However, it should be important to note that a validity study for the POCD conducted in an aged and sick population (duration of type I diabetes ≥ 50 years), determined the sensitivity and specificity of the device to be 80% for both the NCV and SNAP^20^. The authors argued that despite only good reliability and validity, the POCD could be an important tool for feasible routine screening for polyneuropathy, a health care gap not fulfilled by currently available neurography tools^20^.

### Further research

Given that our post-hoc analysis was exploratory using the data from a study primarily designed to answer a different research question^14^, we strongly suggest that prospective long-term, fully crossed, reliability and validity diagnostic studies should be carried out among DPN patients to determine the suitability of the POCD for DPN progression monitoring.

More research would be needed with the aim: (i) to study how the number of “0” measurements can be decreased and/ or interpreted, (ii) to compare the data collected with POCD by non-specialized medical personal (nurses) to data collected by highly specialized medical staff (neurologists), and (iii) to assess the cost-effectiveness of early DPN detection and monitoring in the context of long-term diabetes treatment.

## Conclusions

Despite its easy and pragmatic application, repeated measurements with DPNCheck were necessary to obtain a reliable result. Our results show moderate to good test–retest reliability, but only moderate reliability over time of the device. Since our study was conducted in a sicker population with moderate to severe neuropathy the results question the use of the device to monitor DPNs progression in such patient populations.

### Supplementary Information

Supplementary Tables.

## Data Availability

Data will be available from JD upon reasonable request for scientific purposes.
