# Supplementary material for: Reliability of a novel point of care device for monitoring diabetic peripheral neuropathy
Source: Sci Rep. 2023 Nov 3;13:19043. doi: 10.1038/s41598-023-45841-6 (PMC10624654; doi:10.1038/s41598-023-45841-6)
Supplement: Supplementary file 1 — Supplementary Tables. [file 41598_2023_45841_MOESM1_ESM.docx]

**Appendix. Development and fitting the calculate the ICCs a linear mixed model with fixed and random effects for ICC calculations**

Table A1. The development of the linear mixed model to calculate the one-way random, single measure, absolute agreement ICC [16]

| $y_{ikt}=\mu_{1}+ I_{i}\mu_{2}+\beta_{1}t+ I_{i}\beta_{2}t+\alpha_{\begin{aligned} i1 \\ \end{aligned}}+\alpha_{\begin{aligned} it2 \\ \end{aligned}}+\alpha_{\begin{aligned} k3 \\ \end{aligned}}+\varepsilon_{\begin{aligned} it \\ \end{aligned}}$ |
| --- |
| Where:  ***i*** indicates the study participant  ***k*** indicates the rater  ***t*** indicates the (fixed) session time (week: 0, 8 ,16)  ***I_i_*** is an indicator variable (group assignment: control=0, intervention=1)  ***µ_1_*** is the grand intercept  ***µ_2_*** is the (fixed) intercept intervention effect  ***β_1_*** the (fixed) time trend  ***β_2_*** is the (fixed) time trend intervention effect  ***α_i1_*** is the (random) (time independent) participant effect  ***α_it2_*** is the (random) time-session-specific participant effect  ***α_k3_*** is the (random) (time independent) rater effect  ***ε_it_*** is the error term |
| The process of model fitting: |
| Since only 6 values per patient are available, to prevent overfitting *µ_2,_ β_1_*, *β_2,_ α_2,_* and *α_3_* were considered as optional parameters and for each of the four models (left/right, velocity/amplitude) the model with the highest AIC was chosen by considering all permutations of these parameters. The ICC over measurement sessions was subsequently calculated by adjusting for the chosen fixed variables and then utilizing the variance estimates of the random effects: (Var(***α_i1_***) / {Var(***α_i1_***) + Var(***α_it2_***) + Var(***α_k3_***) + Var(ε_it_)). |

Table A2. Model selection (coefficient and variance estimates) for reliability over measurement sessions

Table A3. Sensitivity analysis for reliability (ICC) with 95% confidence intervals. The analysis excludes all the values of “0” recorded for nerve velocity and amplitude.

|  | Test -retest reliability | | | | Reliability over time |
| --- | --- | --- | --- | --- | --- |
|  | Week 0 | Week 8 | Week 16 | Week 24 |  |
| Velocity [m/s] | | | | |  |
| Right Leg | 0.67  (0.48, 0.82) | 0.86  (0.75, 0.93) | 0.59  (0.33, 0.80) | 0.69  (0.49, 0.83) | 0.71  (0.58, 0.80) |
| Left leg | 0.82  (0.70, 0.90) | 0.73  (0.56, 0.86) | 0.65  (0.45, 0.81) | 0.87  (0.77, 0.94) | 0.61  (0.47, 0.72) |
| Amplitude [µV] | | | | |  |
| Right Leg | 0.84  (0.73, 0.91) | 0.87  (0.77, 0.93) | 0.79  (0.65, 0.88) | 0.82  (0.70, 0.90) | 0.54  (0.37, 0.67) |
| Left Leg | 0.73  (0.57, 0.84) | 0.75  (0.59, 0.86) | 0.79  (0.65, 0.88) | 0.81  (0.68, 0.90) | 0.45  (0.27, 0.58) |
